# Supplementary material for: Species turnover reveals hidden effects of decreasing nitrogen deposition in mountain hay meadows
Source: PeerJ. 2019 Feb 6;7:e6347. doi: 10.7717/peerj.6347 (PMC6368833; doi:10.7717/peerj.6347)
Supplement: Supplemental Information 1 [file peerj-07-6347-s001.pdf]

Appendix A: Comparision of lost/gained species to randomly selected species

Contents

Introduction **1**

Results **1**

Introduction

Temporal turnover is caused by species that are lost between surveys (or are overseen in the second survey) and/or are gained between surveys (or overseen during the first survey). It is likely that different drivers such as Nitrogen deposition or climate warming would cause different species to colonize or disappear from sites. Also methodological issues such as imperfect detection are likely to result in species with particular characteristics that are overseen in one survey and thus result in apparent species gains or losses. We here borrowed ideas from functional ecology in order to try to disentangle the potential drivers of species gains and losses: We compared the characteristics of the species that newly colonized or that disappeared from a site with the same amount of species that were randomly selected from all the species that were observed at the site (i.e. the species assemblage). Instead of the functional traits that are traditionally used in functional ecology such as species height, we used the Ellenberg values that we assumed could best reflect the main drivers that are likely to cause shifts in mountain hay plant communities. These were indicator values for temperature (that are likely to respond to climate warming), soil moisture (likely to respond to changes in precipitation), nutrients (likely to change in response to Nitrogen deposition and land-use intensity) and light (likely to change in response to land-use intensity).

Results

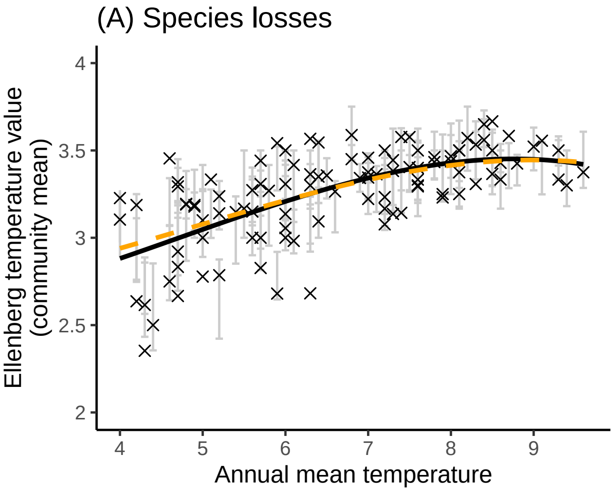

Figure 1: *Average Ellenberg value for* ***temperature*** *of lost (A) and newly appearing (B) species, compared to the same number of species that were randomly selected from all species recorded at the sampling site (i.e. random communities). Crosses indicate the community mean of lost and newly appearing species; the grey vertical lines indicate the range of the 5% and 95% quantiles of community means calculated from the 1000 random communities for each site. The curves show the regression lines with third order polynomials for the community means of lost and newly appearing species (black solid lines) and for the average community means of random communities (orange dotted lines).*

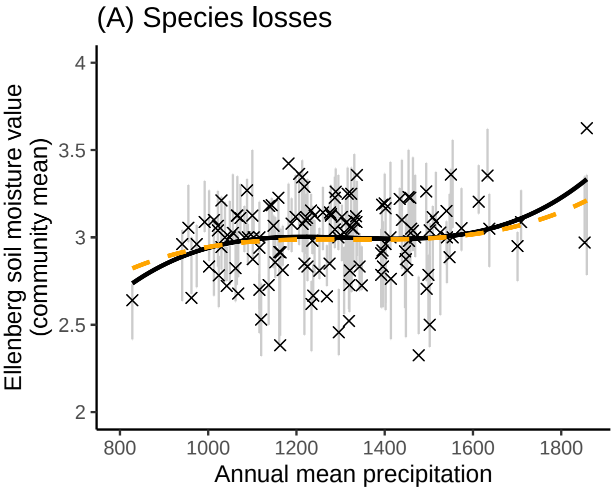

Figure 2: *Average Ellenberg value for* ***soil moisture*** *of lost (A) and newly appearing (B) species, compared to the same number of species that were randomly selected from all species recorded at the sample site (i.e. random communities). Crosses indicate the community mean of lost and newly appearing species; the grey vertical lines indicate the range of the 5% and 95% quantiles of community means calculated from the 1000 random communities for each site. The curves show the regression lines with third order polynomials for the community means of lost and newly appearing species (black solid lines) and for the average community means of random communities (orange dotted lines).*

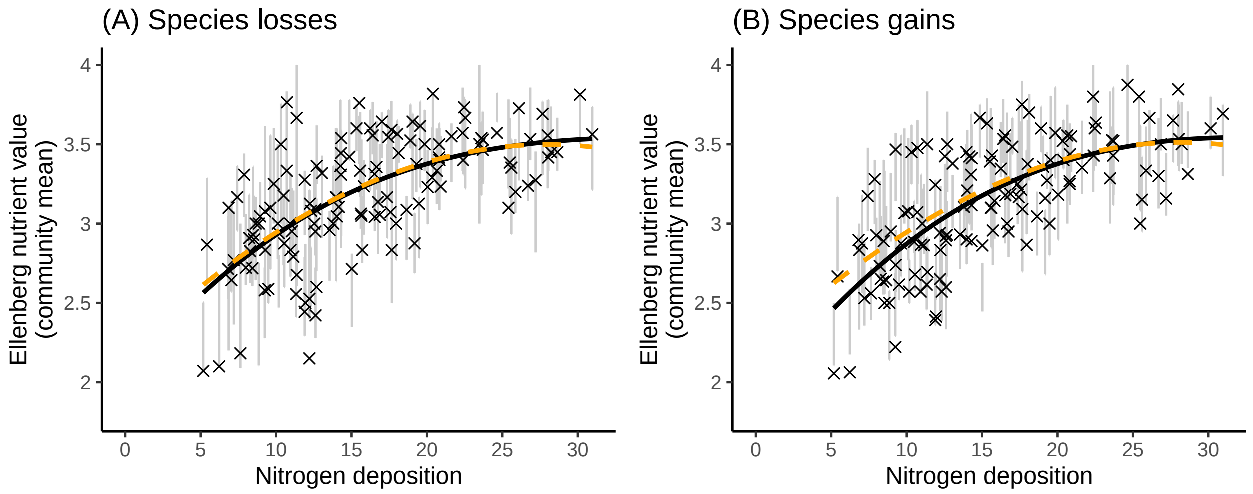

Figure 3: *Average Ellenberg value for* ***nutrients*** *of lost (A) and newly appearing (B) species, compared to the same number of species that were randomly selected from all species recorded at the sample site (i.e. random communities). Crosses indicate the community mean of lost and newly appearing species; the grey vertical lines indicate the range of the 5% and 95% quantiles of community means calculated from the 1000 random communities for each site. The curves show the regression lines with third order polynomials for the community means of lost and newly appearing species (black solid lines) and for the average community means of random communities (orange dotted lines).*

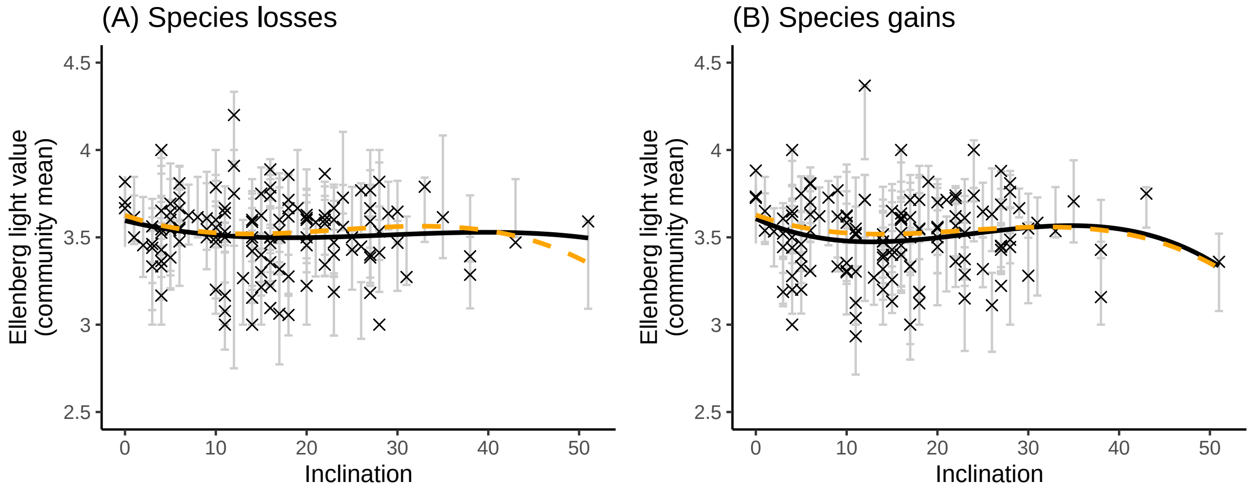

Figure 4: *Average Ellenberg value for* ***light*** *of lost (A) and newly appearing (B) species, compared to the same number of species that were randomly selected from all species recorded at the sample site (i.e. random communities). Crosses indicate the community mean of lost and newly appearing species; the grey vertical lines indicate the range of the 5% and 95% quantiles of community means calculated from the 1000 random communities for each site. The curves show the regression lines with third order polynomials for the community means of lost and newly appearing species (black solid lines) and for the average community means of random communities (orange dotted lines).*
